# Loss of Quiescence in von Hippel-Lindau Hemangioblastomas is Associated with Erythropoietin Signaling

**DOI:** 10.1038/srep35486

**Published:** 2016-10-17

**Authors:** Michael J. Feldman, Saman Sizdahkhani, Nancy A. Edwards, Marsha J. Merrill, Abhik Ray-Chaudhury, Zhengping Zhuang, Russell R. Lonser, Edward H. Oldfield, Prashant Chittiboina

**Affiliations:** 1 Surgical Neurology Branch, National Institute of Neurological Disorders and Stroke, National Institutes of Health, Bethesda, Maryland, USA; 2 Department of Neurological Surgery, Wexner Medical Center, The Ohio State University, Colombus, Ohio, USA; 3 Department of Neurosurgery, University of Virginia Health Sciences Center, University of Virginia, Charlottesville, Virginia, USA

## Abstract

von Hippel-Lindau (VHL) patients develop multiple central nervous system hemangioblastomas (HB). Some HBs become symptomatic with exponential growth or cyst formation following long periods of quiescence. Understanding the factors underlying growth in hemangioblastoma may lead to better strategies to arrest or prevent tumor growth. In 5 VHL patients, we resected quiescent hemangioblastomas (Q-HB) that were en-route during surgical access to symptomatic hemangioblastomas (S-HB), for matched tumor analysis. Quantitative reverse transcriptase analysis demonstrated a 2-fold increase in EPO expression in all S-HB, while 4/5 showed either Hypoxia Inducible Factor-1α or 2α upregulation. Additionally, all S-HB had increased phosphorylated erythropoietin (EPO) receptor and phosphorylated STAT-5 relative to matched Q-HB, with increased phosphorylated JAK-2 largely confined to the stromal cells in clusters within the tumors. These findings suggest that Q-HB to S-HB conversion may be associated with an erythropoietin-signaling loop. Furthermore, we found that EPO is detectable in cyst fluid from S-HB (n = 14), while absent in CSF (n = 1). Additionally, S-HB presentation or S-HB resection does not result in discernible change in serum EPO or hemoglobin (n = 60). These observations suggest that the altered erythropoietin signaling is focal and suggests that studying modulation of erythropoietin receptor pathway may lead to strategies in preventing HB growth.

Von Hippel-Lindau (VHL) disease is an autosomal dominant neoplastic disorder. Majority (80%) of VHL patients develop central nervous system hemangioblastomas (HBs), with tumor development driven by VHL protein (pVHL) loss, resulting in hypoxia inducible factor (HIF) 1α and HIF-2α accumulation^1^,^2^,^3^. In VHL half of the HBs remain quiescent, but the rest display stochastic growth or cyst formation, leading to significant morbidity and mortality^4^,^5^,^6^,^7^. HIF signaling and downstream factors such as VEGF have been implicated in initial tumor development in VHL^8^,^9^,^10^,^11^. HIF-inducible erythropoietin (EPO) and EPO-Receptor (EPO-R) are co-localized in VHL-associated HBs^12^ and renal-cell carcinoma (RCC)^13^ suggesting that autocrine/paracrine EPO/EPO-R signaling loop may underlie RCC formation^14^. But, loss of quiescence in VHL-associated HBs remains unexplained, to a certain extent due to the lack of *in-vitro*/*in-vivo* HB models.

In VHL disease, HBs are resected only when these tumors cause neurologic symptoms^1^,^2^,^6^. Therefore, laboratory investigations involving VHL HBs are exclusively performed on symptomatic HBs (S-HB). Autopsy studies of VHL patients have identified microscopic ‘tumorlets’^11^,^15^, but, no studies have reported on the differences between S-HB and grossly evident quiescent HBs (Q-HB) in surgical patients. In this study, we analyzed differences in tumors from a rare set of five VHL patients who had simultaneously resected S-HB and ‘en-route’ Q-HB.

## Materials and Methods

### Population

HB tumor volumes, serum EPO and hemoglobin (Hgb) were measured in VHL subjects with HBs (NCT00005902, NIH 00-N-0140). S-HB tumors were resected (by EHO and PC) in an ongoing clinical trial (NCT00060541, NIH 03-N-0164), for symptomatic tumor growth or cyst formation^6^. Quiescent HBs (Q-HBs) were resected when encountered en-route during surgical access to S-HB. S-HB with cysts were prospectively selected for intraoperative cyst fluid aspiration and collection prior to surgical resection of HBs. Cyst fluid was immediately frozen at −80 °C for later use. Enzyme-linked immunosorbent assay (ELISA) was used to measure EPO level in S-HB cyst fluid (n = 14) and available paired CSF (n = 1). Clinically reported serum EPO (118 subjects) and hemoglobin values (60 subjects) were analyzed for changes related to S-HB resection. The trials are approved by the Combined Neuroscience Institutional Review Board of the National Institutes of Health, Bethesda, MD, USA. The trials were conducted in accordance with the approved guidelines. All subjects provided informed consent for trial participation, and for use of clinical data and samples.

### Expression analysis

Primers for HIF-1, HIF-2, glucose transporter 1 (GLUT-1), vascular endothelial growth factor (VEGF) (Qiagen), and EPO (BioRad) were used to perform qRT-PCR on tumor RNA.

### Western blot

Anti-STAT5 (Santa Cruz sc-835), anti-phosphorylated STAT5 (Millipore 05-495), anti-EPO-R (Santa Cruz sc-697), anti-phosphorylated EPO-R (Abcam ab79824), and anti-actin (Sigma) probes were used and fluorescently conjugated secondary antibodies used to image on Odyssey CLx (Li-Cor).

### Immunohistochemistry

Formalin fixed HBs were stained with anti-phosphorylated JAK2 using Leica BondMax and reviewed by blinded neuropathologist (ARC).

## Results

**Figure 1:**
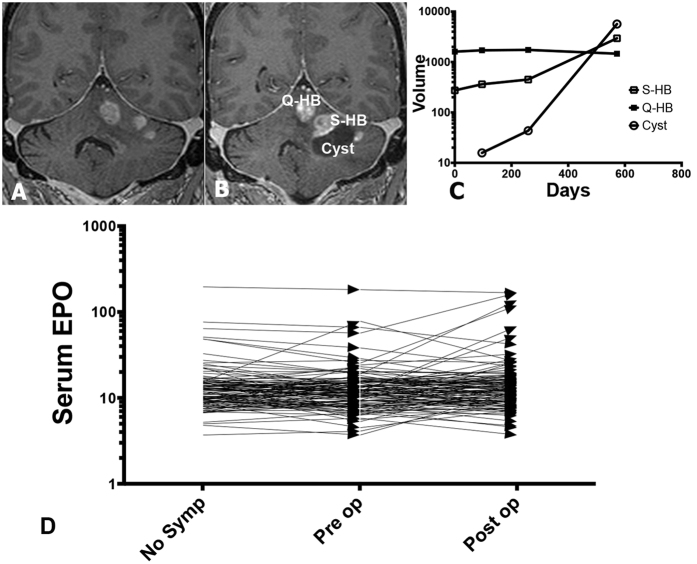
Symptomatic hemangioblastomas are not associated with a change in serum erythropoietin. Surveillance coronal gadolinium contrast enhanced T1 MRI images of patient 4 obtained 42 months prior to surgery (**A**) and at symptom presentation (**B**) demonstrate an increase in tumor volume (S-HB, 2,957 mm^3^) as well as formation of a new cyst (5,682 mm^3^). There was no significant change in the volume of the adjacent quiescent hemangioblastoma (Q-HB, 1,463 mm^3^) (**C**). In C, y-axis represents volume in cubic millimeters plotted on a log10 scale. The x-axis represents time in days. D: Pre-operative and post-operative serum erythropoietin (EPO) levels (mIU/mL) of 117 VHL patients that underwent surgical resection of symptomatic hemangioblastomas are plotted in D. No trend in the change of serum EPO levels was evident following surgical resection of symptomatic hemangioblastomas. The y-axis represents serum EPO levels plotted on a log10 scale. EPO–erythropoietin, Q-HB–quiescent hemangioblastoma, S-HB–symptomatic hemangioblastoma.

Clinical and imaging characteristics of the 5 patients are summarized in Table 1. In all cases, the location of Q-HB was adjacent to the S-HB, and within the planned surgical approach. The clinical presentation of S-HB depended on its location (Table 1), and was either due to tumor volume increase, cyst formation or both (Fig. 1). In contrast, Q-HBs demonstrated minimal growth, and no cyst formation (Supplementary Figure 1). No significant changes in serum Hemoglobin (Supplementary Figure 2) or serum EPO (Fig. 1) were detected with S-HB presentation or following resection of S-HB.

**Figure 2:**
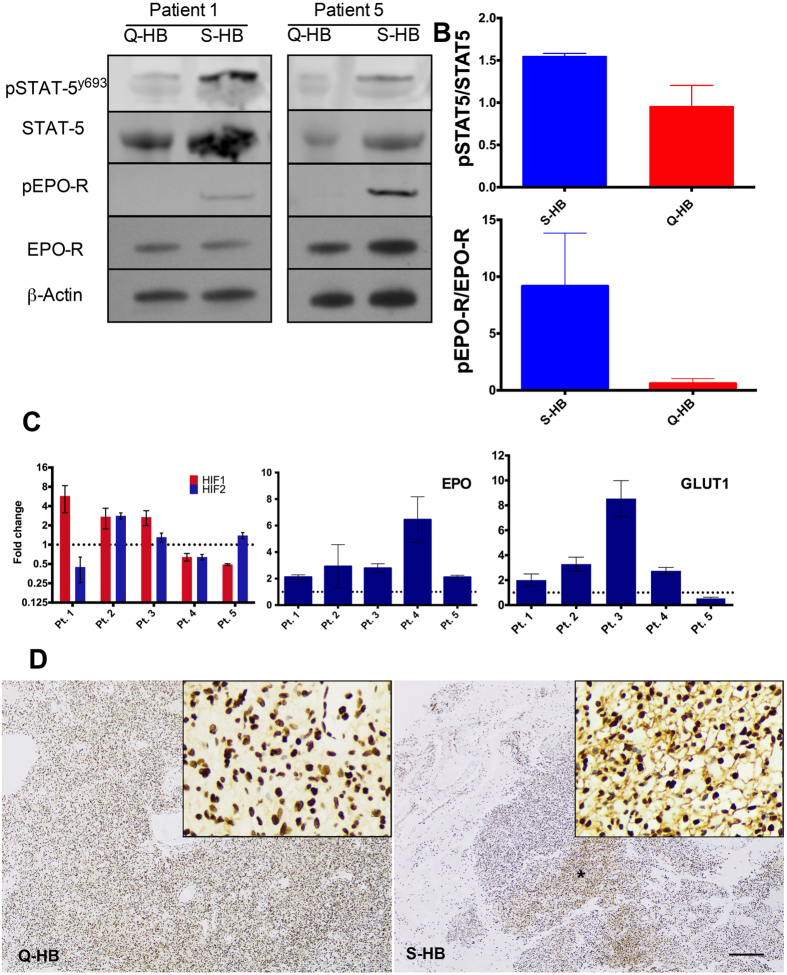
EPO-HIF signaling in Symptomatic and Quiescent Hemangiblastomas. (**A**) Increased phosphorylated EPO-receptor (pEPO-R) observed in representative sytmptomatic hemangioblastomas (S-HB) when compared with quiescent hemangioblastomas (Q-HB), indicative of increased EPO-R activation by EPO. Increased tyrosine phosphorylated STAT-5 in symptomatic HBs relative to quiescent HBs indicative of increased transduction of EPO-R activation in S-HBs. (STAT-5 and pSTAT-5 detected within the same experiment. EPO-R and pEPO-R detected within other experiments conducted under similar conditions (Supplementary Data available on request). Images were cropped and presented together for clarity.) (**B**) Densitometry revealed trend towards increased ratio of both pSTAT5 and pEPO-R in S-HB (n = 5). (**C**) HIF-1α and HIF-2α variably expressed, with overexpression of HIF-1α or HIF-2α in 4/5 S-HBs in comparison to matched Q-HBs. EPO expression increased in all S-HBs relative to Q-HBs, and GLUT1 expressed at higher levels in 4/5 S-HBs than Q-HBs, suggesting some role of aberrant HIF signaling associated with S-HB growth. Expression values are relative to levels in Q-HB in the same patient. (**D**) Phosphorylated JAK-2 in focal areas exhibited denser staining in S-HB, as seen here in a representative selection of a focal region from a S-HB and a typical region of Q-HB from Patient 3. Jak-2 phosphorylation transduces the EPO-R signal to STAT-5, completing a signaling loop from EPO expression to EPO-R activation and STAT-5 phosphorylation. The focality of this staining also is consistent with serum EPO findings (as shown in Fig. 1D), suggesting a paracrine/autocrine effect, rather than effects of elevated systemic EPO0. EPO–erythropoietin, EPO-R–erythropoietin receptor, GLUT1–glucose transporter 1, HIF–hypoxia inducible factor, Q-HB–quiescent hemangioblastoma, S-HB–symptomatic hemangioblastoma, STAT5–signal transducer and activator of transcription 5.

HBs expressed EPO-R variably, but S-HB had consistently elevated tyrosine phosphorylated EPO-R (densitometry mean absorption values: p = 0.11, 95% CI −46.67 to 29.54) (pEPO-R). STAT5 expression was unchanged between S-HBs and Q-HBs, but tyrosine phosphorylated STAT5 (STAT5^y694^) was elevated in S-HBs (densitometry mean absorption values: p = 0.025, 95% CI −1.18 to 0.00) (Fig. 2). Though a myriad of factors can induce STAT5 tyrosine phosphorylation, the association with increased pEPO-R is suggestive of an EPO-driven mechanism for STAT5 activation to a greater extent in S-HBs than Q-HBs^1^.

IHC probing of phosphorylated-JAK2 demonstrated mild membranous staining in both Q-HBs and S-HBs, with focal areas of strong membranous staining (Fig. 2) more common in S-HBs than Q-HBs. Importantly, the presence of focal stromal cell membranous pJAK2 in localized clusters of tumor cells with increased levels of EPO-R activation suggests paracrine effects of EPO in regions of local activation^6^.

qRT-PCR was used to elucidate the role of HIF pathway in HB growth. EPO was reliably upregulated (mean fold change 2.11 to 6.45, all p < 0.05) in S-HBs relative to Q-HBs. GLUT-1 expression was increased (mean fold change 2.00 to 8.54, all p < 0.05) in 4 of 5 S-HBs. HIF-1α and HIF-2α expression changes varied between patients; 3/5 patients had increases (mean fold change 2.68 to 5.74) in HIF-1α, and 3 patients had HIF-2α increased expression (mean fold change 1.30 to 2.82) in S-HBs relative to Q-HBs (Fig. 2).

HIF-2α has been demonstrated to be a transcriptional target of STAT5, and STAT5 activation can drive glucose uptake and metabolic supply in hematopoietic cells^16^. The upregulation of the STAT5 target GLUT1 also suggests STAT5-activation mediating selective HIF cascade activation. HIF-2α, in contrast to HIF-1α, signaling is a significant regulator of EPO^17^. Thus the increased EPO-R signaling with STAT-5 induced HIF-2α and resultant EPO upregulation and activation seen in focal stromal cells suggests an EPO-HIF-2α signaling loop driving local tumor evolution.

As shown before^15^,^18^, S-HB associated cyst fluid samples (n = 14) had detectable levels of EPO (9.98 ± 10.10, range 1.72–33.9 pg/mL). Paired VHL CSF sample had undetectable EPO level (Supplementary Figure 2). We found no change in serum EPO levels leading up to symptomatic clinical presentation (Mean difference −0.514 mU/mL, 95% CI −2.044 to 1.023 mU/mL, p = 0.51) in 117 patients with VHL disease (Fig. 1D). Additionally, surgical removal of S-HB did not result in a significant change in serum EPO levels (Mean difference 1.774 mU/mL, 95% CI −8.931 to 12.48, p = 0.74) (Fig. 1D). The lack of increased serum EPO from S-HBs suggests localized EPO signaling (autocrine/paracrine) driving focal areas of tumor proliferation through induced HIF-2α. Similarly, polyglobulia^16^ was notably absent with S-HB presentation, and S-HB resection did not result in fall of Hgb (ANOVA, p = 0.11) (Supplementary Figure 2). Taken together, these findings indicate that S-HB may release EPO focally (cyst fluid, but not in CSF), with no discernible systemic effects (change in serum EPO and Hgb).

## Discussion

In this study the differences in EPO and HIF mediated signaling pathways were analyzed in a unique cohort of 5 patients with simultaneously resected Q-HBs and S-HBs. All 5 patients showed higher EPO expression in the S-HB relative to the Q-HB, with 4 of 5 showing the same pattern in GLUT1 expression but no other notable changes in HIF signaling cascade observed. EPO upregulation was associated with increased EPO-R signaling, as seen by the increased relative pEPO-R and downstream induction of pSTAT5 in S-HBs relative to Q-HBs. This signaling was confined to the stromal cells, and was focally present in S-HBs as seen through membranous JAK2 phosphorylation on IHC. GLUT1 and STAT5 target genes were also upregulated in S-HBs, indicating active STAT5 nuclear signaling. These results may suggest an EPO-STAT5 autocrine/paracrine loop in HB loss of quiescence with increased activation of EPO-R, increased tyrosine phosphorylation of STAT5 and upregulation of downstream GLUT1 in S-HBs. Increased EPO-signaling activity is also suggested by the increased pEPO-R in S-HBs as the JAK-2 mediated STAT5 phosphorylation would also auto-phosphorylate both JAK2 and EPO-R^17^. The absence of detectable EPO on patient-wide analysis also suggests that this is an autocrine/paracrine effect. It is important to note that our findings are limited by the inability to assess this mechanism *in-vitro*, as no viable cell model exists for VHL-associated HBs. Further, due to the scarcity of these paired samples, both the quality and quantity of the analyzable tissue accrued over the past 10 years was extremely variable, and we were limited in our ability to explore more possible pathways in multiple patients in this study by a lack of remaining archived tumor specimen. Further, though our posited mechanism of an EPO and HIF mediated loop would explain variable growth between Q-HBs and S-HBs, and local secretion of EPO from HBs also fits with the focally observed pJAK2 on IHC, we are currently unable to offer concrete evidence of a “trigger” that could drive individual stromal cells (or clusters of stromal cells) into this vicious EPO-mediated cycle. However, our findings are only the first step towards understanding what drives quiescent HBs into growth and may offer novel ways to both detect and prevent growth in VHL-associated hemangioblastomas.

## Additional Information

**How to cite this article**: Feldman, M. J. *et al.* Loss of Quiescence in von Hippel-Lindau Hemangioblastomas is Associated with Erythropoietin Signaling. *Sci. Rep.* **6**, 35486; doi: 10.1038/srep35486 (2016).

## Supplementary Material

Supplementary Information

## Figures and Tables

**Table 1:** Clinical and radiologic features of the 5 patients included in the current study.

Patient No.	Sex	Age in years	Surgery Date	Presenting symptoms	S-HB Location	S-HB Volume (mm^3^)	S-HB Associated Cyst Volume (mm^3^)	Q- HB Location	Q-HB Volume (mm^3^)	Q-HB Associated Cyst Volume (mm^3^)
1	F	57	3/19/03	Progressive Ataxia	Dorsal C-4	107	7,835	Dorsal C-3	358	0
2	F	60	8/24/04	Progressive Ataxia	Ventral C-7	545	0	Right C-5 nerve Root	41	0
3	M	26	7/3/06	Headache	Right cerebellar hemisphere	66	18,969	Dorsal cervico-medullary junction	3,211	0
4	M	49	1/28/14	Altered mental status, left dysmetria	Left cerebellar hemisphere	2,957	5,682	Superior vermis	1,463	0
5	M	39	11/5/14	Gait imbalance, dysmetria, right arm weakness	Cerebellar vermis	7,724	0	Right cerebellar hemisphere	1,477	0

The spinal locations of the hemangioblastomas are represented by the vertebral levels (C–cervical). Volumes are represented in mm^3^. Q- HB quiescent hemangioblastoma, S- HB symptomatic hemangioblastoma.
