# Supplementary material for: Loss of Quiescence in von Hippel-Lindau Hemangioblastomas is Associated with Erythropoietin Signaling
Source: Sci Rep. 2016 Oct 17;6:35486. doi: 10.1038/srep35486 (PMC5066214; doi:10.1038/srep35486)
Supplement: Supplementary Information [file srep35486-s1.pdf]

Loss of Quiescence in von Hippel-Lindau Hemangioblastomas is Associated with Erythropoietin Signaling

Michael J. Feldman[1], Saman Sizdahkhani[1], Nancy A. Edwards[1], Marsha J. Merrill[1], Abhik Ray-Chaudhury[1], Zhengping Zhuang[1], Russell R. Lonser[1,3], Edward H. Oldfield[1,2], and Prashant Chittiboina[1]

1.Surgical Neurology Branch, National Institute of Neurological Disorders and Stroke, National Institutes of Health, Bethesda, Maryland, 2.Department of Neurosurgery, University of Virginia Health Sciences Center, University of Virginia, Charlottesville, Virginia, 3.Department of Neurological Surgery, Wexner Medical Center, The Ohio State University, Colombus, Ohio

Supplementary figures and supplementary data

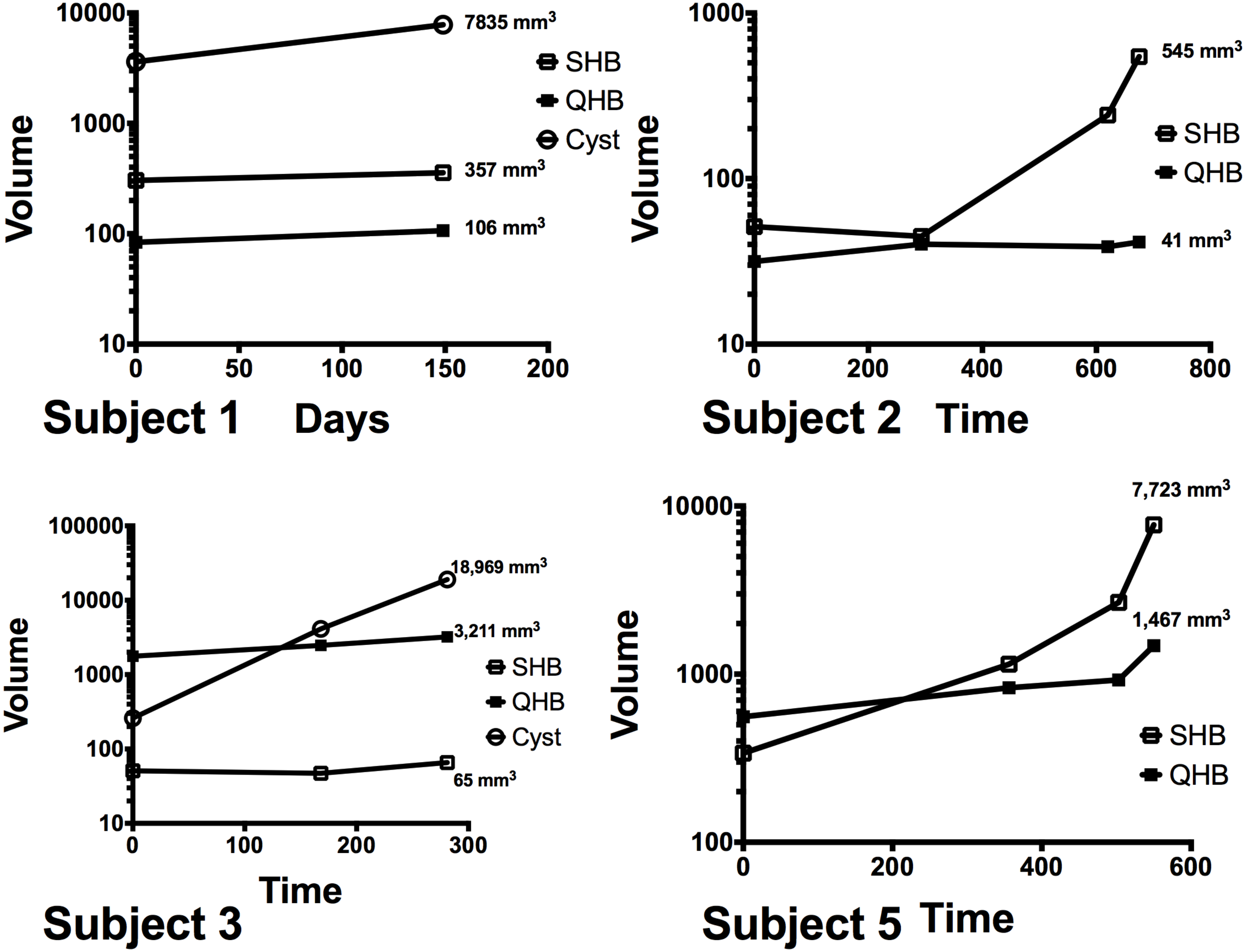

Supplementary Figure 1: Serial volumetric measurements of symptomatic hemangioblastoma (S-HB), S-HB associated cysts and quiescent hemangioblastomas (Q-HB) in four subjects (1, 2, 3 and 5). The x-axis represents time in days. The y- axis represents volume in cubic millimeters on a log 10 scale.

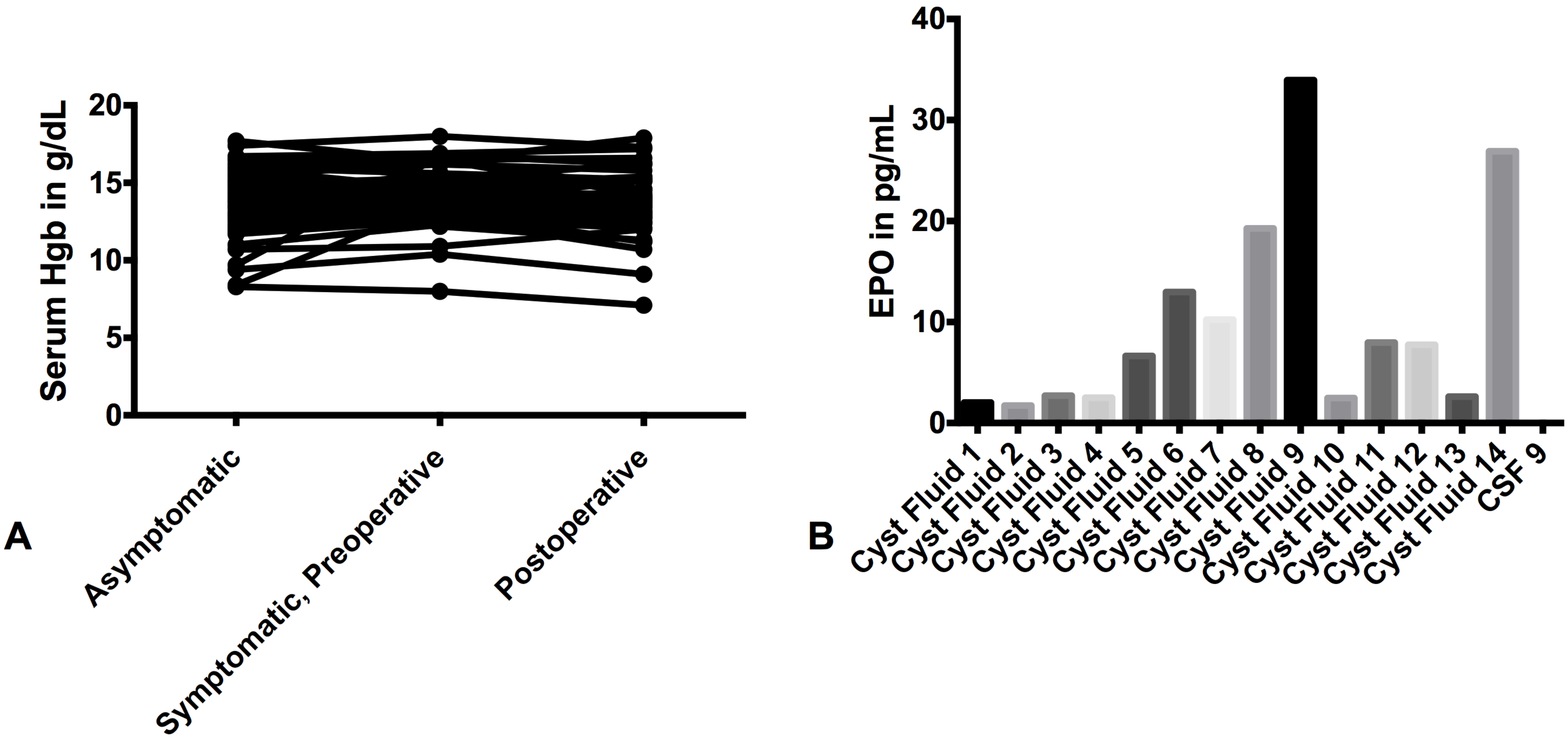

Supplementary Figure 2:
Serum Hemoglobin (Hgb) was measured in 60 subjects (A) at least 6 months to 1 year prior to presentation (asymptomatic)with symptomatic hemangioblastoma (S-HB). These values were then compared with Hgb measured within a month prior to surgery (symptomatic, pre-operative) and values following convalescence from surgery at least 6 months following surgery (postoperative). No major trends in Hgb change was detected with presentation or resection of S-HB.

Prospectively collected cyst fluid erythropoietin (EPO) measurements revealed detectable EPO in cyst fluids of S-HB, but, no CSF was detected in the CSF of one subject that was collected during resection of S-HB (B).

Supplementary Data

Western Blots

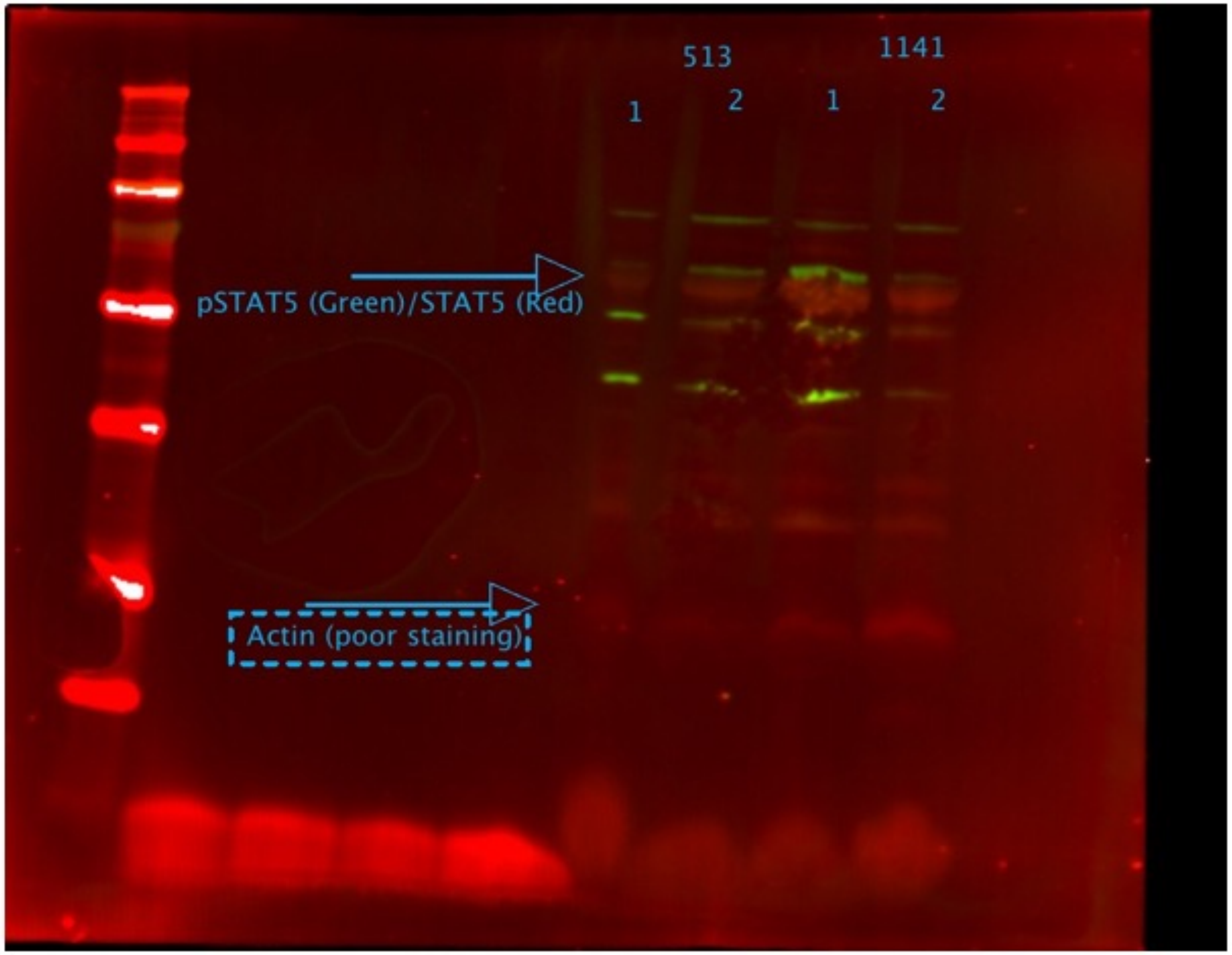

STAT-5 and p-STAT-5 detected within the same experiment in tumor lysates from two tumors (Pt 5, #513 and Pt 1, #1141). Uncropped lanes and bands highlighted on Li-Cor readout.

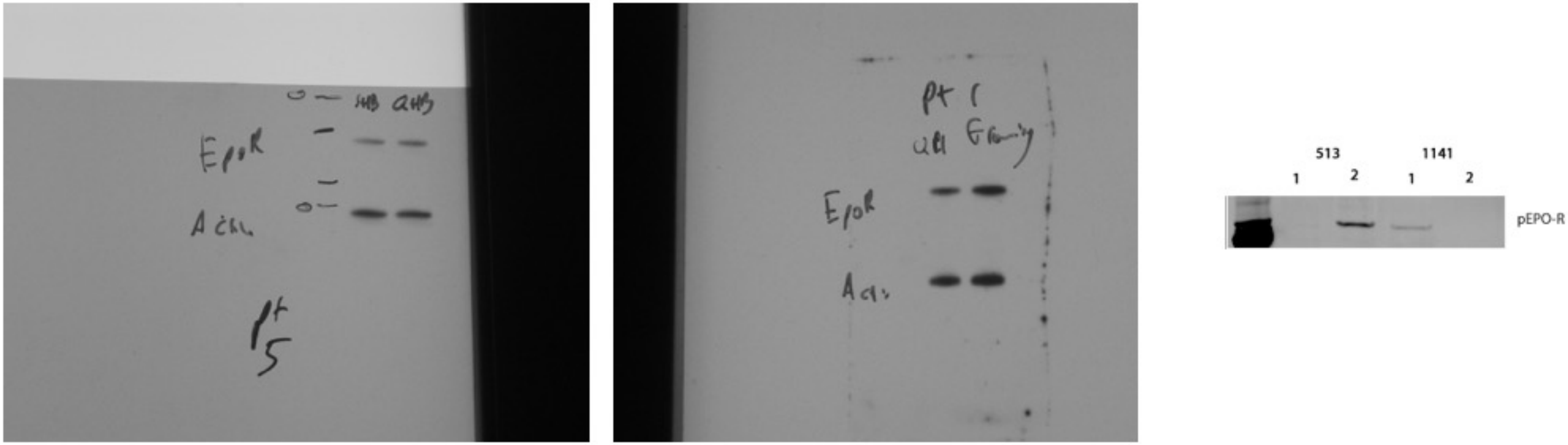

EPO-R and pEPO-R were detected in tumor lysates from two tumors (Pt1, #1141 and Pt5, #513).
